# Human Cancer and Platelet Interaction, a Potential Therapeutic Target

**DOI:** 10.3390/ijms19041246

**Published:** 2018-04-20

**Authors:** Shike Wang, Zhenyu Li, Ren Xu

**Affiliations:** 1 Markey Cancer Center, University of Kentucky, Lexington, KY 40536, USA; Shike.Wang@uky.edu; 2 Division of Cardiovascular Medicine, Department of Internal Medicine, College of Medicine, University of Kentucky, 741 South Limestone Street, Lexington, KY 40536, USA; zhenyuli08@uky.edu; 3 Department of Molecular and Biomedical Pharmacology, University of Kentucky, Lexington, KY 40536, USA

**Keywords:** cancer metastasis, platelet, biomarker, cancer therapy

## Abstract

Cancer patients experience a four-fold increase in thrombosis risk, indicating that cancer development and progression are associated with platelet activation. Xenograft experiments and transgenic mouse models further demonstrate that platelet activation and platelet-cancer cell interaction are crucial for cancer metastasis. Direct or indirect interaction of platelets induces cancer cell plasticity and enhances survival and extravasation of circulating cancer cells during dissemination. In vivo and in vitro experiments also demonstrate that cancer cells induce platelet aggregation, suggesting that platelet-cancer interaction is bidirectional. Therefore, understanding how platelets crosstalk with cancer cells may identify potential strategies to inhibit cancer metastasis and to reduce cancer-related thrombosis. Here, we discuss the potential function of platelets in regulating cancer progression and summarize the factors and signaling pathways that mediate the cancer cell-platelet interaction.

## 1. Introduction

During tumor progression, a small number of cancer cells invade into surrounding tissue from the primary lesion and get into the circulation system through the intravastation process [1]. These circulating tumor cells (CTCs) were first identified by Thomas Ashworth in 1869 [2]. Given the recent progress in CTC isolation, the association between CTC and cancer metastasis or prognosis has been identified in many types of cancer, including lung cancer [3,4], breast cancer [5], colon cancer [6] and castration-resistant prostate cancer [7]. In fact, multiple clinical trials have been done or are ongoing to test whether CTC counts can be used as a prognosis marker. The roles of CTCs in cancer metastasis and cancer relapse are well established in animal models [8,9]. Single cell RNA sequencing data show that CTCs exhibit the epithelial-to-mesenchymal transition (EMT) [10] and stem cell phenotypes [11,12], suggesting that CTCs are the driver of cancer metastasis.

CTCs directly interact with red blood cells [13], platelets, macrophages [14], and many other immune cells [15,16,17]. CTCs also encounter shear stress induced by blood flow [18]. These interactions play important roles in the colonization of CTC at distant organs. It has been shown that CTCs induce the differentiation of macrophages. Cytokines secreted by the differentiated macrophage, in turn, enhances CTC-inflammatory cell interaction, stroma breakdown, and CTC invasion [19,20]. Clinical data show that the number of CTC is negatively associated with CD3^+^ T cells and cytotoxic (CD8^+^) T cells [21], suggesting that T cell-mediated immunity is abnormal in patients with high CTC counts [16]. In addition, programmed death-ligand 1 (PD-L1) expression has been detected on the surface of CTCs, which may contribute to the immune escape from T cells and promote cancer metastasis [22].

Clinical evidence and mouse models demonstrate that platelet-cancer cell interaction is crucial for cancer metastasis [23]. Platelets, originally derived from megakaryocytes in the bone marrow [24], are the key regulator of thrombosis [25,26]. The major function of platelets is to prevent bleeding and reduce blood loss in case of vascular injury [27]. It has been reported that platelet count is associated with metastasis and poor prognosis in cancer patients [28,29]. Consistently, with the clinical evidence, the size and number of tumor nodules are reduced by halving the platelet count in the murine model of ovarian cancer [30]. In addition, long-term application of low-dose anti-platelet drugs, such as aspirin, inhibits cancer metastasis and significantly reduces cancer incidence [31,32]. Together these results suggest that platelet activation is a potential target and prognosis marker for cancer treatment [29,33,34]. 

In this review, we discuss the function and regulation of cancer cell-platelet interaction during cancer development and progression. We also summarize the factors and pathways mediating the interaction and potential targets to halt platelet-induced cancer progression.

## 2. Roles of Platelets in Cancer Development and Progression

### 2.1. Roles of Platelets in Tumor Development

Platelet activation by physiological agonists results in secretion of a variety of cytokines and growth factors in the platelet releasates (molecules released after platelets activating) [35,36]. Platelet releasates, induced by the agonists of the thrombin receptors, protease activated receptor-1 (PAR1) and PAR4 [37], promote the proliferation of MCF-7 and MDA-MB-231 breast cancer cells and angiogenesis via the phosphoinositide 3-kinase/protein kinase C (PI3K/PKC) pathway [38]. Platelet activation induced by other agonists, including the adenosine diphosphate (ADP) (through its receptor P2Y12 and P2Y1) also promotes tumor growth in ovarian cancer and pancreatic cancer [39,40]. Recently, the relationship between P2Y12 and cancer was reviewed by Ballerini et al. indicating the important role of P2Y12 in malignant cells [41].

Many of the platelet-derived factors involved in cancer progression are important components of tumor microenvironment, such as transforming growth factor beta (TGF-β), vascular endothelial growth factor (VEGF), and platelet-derived growth factor (PDGF) [42,43,44]. TGF-β1, a member of the TGF-β family, can be secreted during platelets activation [45]. A recent study showed that platelet-derived TGF-β1 promotes the growth of primary ovarian cancer in murine models [46]. Incubation of platelets with TGF-β1-blocking antibody or downregulation of TGF-βR1 receptor expression in cancer cells with siRNA inhibits proliferation in ovarian cancer cells [47]. It has been shown that platelet extracts induce hepatocellular carcinoma growth [48] by suppressing the expression of Krüppel-like factor 6 [49], a tumor suppressor in many cancers [50]. Protein levels of VEGF, PDGF and platelet factor 4 (PF4) in platelets are elevated in colorectal cancer patients compared to healthy control [51]. VEGF and PDGF are the well-characterized angiogenesis regulator [52,53]. It has been shown that platelets induce tumor angiogenesis by releasing platelet-derived growth factor D and VEGF, and subsequently promotes the tumor growth [54]. PF4 accelerates Kras-driven tumorigenesis in lung cancer [55]. Interestingly, PF4 has also been identified as a chemokine that exhibits anti-angiogenesis activity [56] and may inhibit tumor growth through anti-angiogenesis [57]. PF4 may bind to VEGF or basic fibroblast growth factor (bFGF), thereby inhibiting receptor binding and bFGF dimerization [58,59]. These results suggest that the function of PF4 in cancer development is context-dependent.

Platelet-derived microRNA has recently been identified as a regulator of tumor development. Lawrence E. Goldfinger showed that platelet-derived microparticles transfer miR-24 into cancer cells. Platelet miR-24 subsequently targets mt-Nd2 and Snora75, modulates mitochondrial function, and inhibits tumor growth [60]. Although most data support that platelets promote cancer progression, especially in metastatic dissemination [61,62], this study suggest that the platelets suppress tumor development at the initiation stage. Therefore, the function of platelets in cancer progression may be stage- and context-dependent. 

### 2.2. Roles of Platelets in Cancer Metastasis

About 90% cancer related death is due to cancer metastasis [63]. Depletion of platelets or inhibition of platelet activation inhibits cancer metastases [64,65], indicating that platelets are required for cancer metastasis. During metastasis, cancer cells must detach from the primary tumor and intravasate into circulation, where tumor cells encounter immune cells and experience fluid shear stress. The shear force can sensitize both colon and prostate cancer cells to TNF-related apoptosis-inducing ligand (TRAIL)-induced apoptosis [66]. It has been speculated that binding of platelets to cancer cells protects cancer cells from shear-induced damage and facilitates cancer colonization [67].

EMT, characterized by disruption of cell-cell adhesion and expression of mesenchymal markers, provides cancer cells with the increased cell plasticity and stemness required for colonization and metastasis [68,69]. Platelet-cancer cell interaction promotes EMT in tumor cells and enhances the rate of tumor extravasation in vivo through the TGF-β/Smad and NF-κB pathways [70,71]. Platelet microparticles (PMPs) are the most abundant microparticles in the blood, which may transport miRNA and many other factors promoting EMT. For instance, miR-939 in PMPs promotes the EMT by downregulating E-cadherin and claudin by targeting the 3′UTR region of these genes [72]. In addition, the platelet receptor C-type lectin-like receptor 2(CLEC2) binds to Aggrus expressed in cancer cells and induces the EMT phenotype and cancer metastasis [73]. Tissue factor (TF) is a transmembrane receptor that initiates the extrinsic coagulation pathway. TF is highly expressed in many cancers, and the expression is associated with cancer metastasis [74]. Co-culturing patient-derived ovarian cancer cells with platelets increases the EMT/stemness biomarker and TF protein levels in cancer cells. TF further enhances platelet recruitment and tumorsphere formation [75]. Platelet-released PDGF can also enhance Cyclooxygenase (COX)-2 expression and induce the EMT markers [76]. These studies suggest that platelets promote the EMT process through multiple pathways.

Platelet activation and adhesion depend on integrin signaling [77]. Five integrins, including α2β1, α5β1, α6β1, αIIbβ3 and αvβ3, have been identified in platelets, which bind preferentially to collagen, fibronectin, laminin, vitronectin, and fibrinogen, respectively [78]. It has been shown that platelet α6β1 mediates the platelet-cancer cell interaction by binding to metalloproteinase (ADAM) 9 on tumor cells, and subsequently induces platelet activation and cancer cell extravasation. Deletion of integrin α6β1 on platelets reduces lung metastasis [79]. Knockout mouse experiments show that platelet β3 integrin contributes to cancer bone metastasis [80]. Treatment with the αIIbβ3 antagonist significantly reduces the bone metastasis of breast cancer in mice though depletion of platelet derived lysophosphatidic acid (LPA) [81]. Interestingly, αIIbβ3 expression is also detected on tumor cells [82]; however, roles of the tumor cell αIIbβ3 in cancer metastasis remains unclear.

Anoikis is a programmed cell death induced by cell detachment [83]. Anoikis resistance is required for CTC survival and colonization in distant organs. Platelet interaction protects cancer cells from anoikis [84]. RhoA-(myosin phosphatase targeting subunit 1) MYPT1-protein phosphatase (PP1)-mediated Yes-associated protein 1 (YAP1) dephosphorylation and nuclear translocation are induced by platelets, resulting in apoptosis resistance [85]. Apoptosis signal-regulating kinase 1 (Ask1) is a stress-responsive Ser/Thr mitogen-activated protein kinase kinase kinase (MAP3K) in the Jun N-terminal kinases (JNK) and p38 pathways [86]. Once the Ask1 is deficient in platelet, activating phosphorylation of protein kinase B (Akt), JNK, and p38 is reduced, and tumor metastasis is attenuated [87].

Acid sphingomyelinase (Asm) is another secreted protein mediating the interaction between cancer cells and platelets. Asm released from activated platelets induces the production of ceramide. Ceramide activates the α5β1 integrin on melanoma cells and promotes metastasis in vivo [88]. Treatment with exogenous Asm activates p38 kinase pathway in melanoma cells. Activation of p38 is required for tumor cell adhesion and metastasis in vivo [89]. This evidence suggests that platelets promote cancer metastasis through direct and indirect interactions.

### 2.3. Impact of Platelet on the Anti-Tumor Immunity

In order to survive in circulation, CTCs need to overcome not only the shear force-induced damage, but also attacks from immune cells. Antitumor immunity activity is well-characterized in NK cells [90]. Depletion of NK cells significantly promotes cancer metastasis in mouse [91]. It has been shown that binding of platelets protects CTCs from NK cells [92]. MHC class I is usually downregulated in tumor cells [93]. Platelet-derived MHC class I is transferred to tumor cells upon interaction, subsequently reducing the NK cells’ antitumor reactivity [94]. In addition, TGF-β released by platelets inhibits the anti-tumor activity of NK cells by reducing the expression of natural-killer group 2, member D on NK cells [95].

Platelet-derived TGF-β has multiple functions in suppressing the antitumor immunity. TGF-β1 is required for converting conventional CD4^+^ T (Tconv) cells into induced regulatory T (iTreg) cells [96]. In the hemophilia A mice, TGF-β1 along with other platelet contents induces Foxp3 expression in Tconv cells, and then converts them into functional iTreg cells [97]. Treg cells have the ability of killing activated T cells through a granzyme B (GzmB)-dependent mechanism [98,99]. In addition, platelets constitutively express the non-signaling TGF-β-docking receptor glycoprotein A repetitions predominant (GARP) [100]. Platelet-intrinsic GARP may facilitate TGF-β activation in tumor tissue and subsequently constrains the T cell function in the cancer microenvironment [101]. These data support the hypothesis that platelets promote cancer metastasis by repressing immune response.

## 3. Cancer Induces Platelet Activation

The interaction between platelets and cancer cells is bidirectional, and cancer cells have profound effects on platelet generation and activation. Cancer patients often have an abnormal platelet count and activation. More than five-fold increase of thrombosis and thromboembolism incidences have been detected in cancer patients compared with normal person [102]. Furthermore, the extracellular vesicles derived from breast cancer cell lines induce tissue factor-independent platelet activation and aggregation, providing a potential mechanism for cancer-associated thrombosis [103]. Fiorella Guadagni et al. showed that the cancer-associated oxidative stress also contributes to persistent platelet activation [104].

Cathepsins K (CAT K) is a protease up-regulated in many cancers [105,106]. It has been shown that platelet aggregation is induced by CAT K in a dose-dependent manner through proteolytically-activated receptors (PAR) 3 and 4. During this process, sonic hedgehog, osteoprotegerin, parathyroid hormone-related protein, and TGF-β are released, which, in turn, induce downstream signaling pathways in breast cancer cells [107]. This study further suggests that cancer cells have profound impacts on platelet activation.

Levels of lipid phosphate phosphatase 1 (LPP1), the key enzyme in phospholipid biosynthesis pathways, is reduced in platelet derived from ovarian cancer patients. The reduction of LLP1 may contribute to the increased risk of thrombosis in cancer patient [108]. However, plasma levels of β-thromboglobulin and PF-4, two markers of platelet α granule secretion and platelet aggregation, have little difference between ovarian cancer patient and patients with benign ovarian tumors. In addition, platelets derived from ovarian cancer patients do not exhibit an enhanced aggregation response to ADP or collagen [109]. These findings suggest that platelet hyperactivation in cancer patients is cancer-type dependent.

## 4. Platelets, a Potential Therapeutic Target and Biomarker for Cancer Treatment

### 4.1. Platelet Is a Potential Target to Suppresses Cancer Metastasis

Given the crucial roles of platelets in cancer progression, targeting cancer cell-platelet interaction is considered a promising strategy for cancer prevention and treatment. In fact, many compounds that target platelets exhibit anti-tumor activities. Aspirin is the traditional drug to reduce fever, pain, and inflammation [110]. It is also widely used in patients with a high risk of heart disease and thrombosis because of its unique ability to inhibit platelet COX-1 [111]. Treatment with aspirin suppresses the function of platelets in promoting cancer metastasis in mice [112]. Population and clinical studies also demonstrate that aspirin significantly reduces the risk of colon cancer development and inhibits cancer growth and invasiveness [113,114]. Tamoxifen is used widely as antiestrogen therapy for breast cancer [115]. Interestingly, one study shows that tamoxifen and its metabolite, 4-hydroxytamoxifen, directly inhibit platelet-mediated metastasis [116]. Specifically, delivery of the platelet aggregation inhibitor ticagrelor to tumor tissue also inhibits the EMT phenotypes and cancer metastasis in vivo [117].

The compound 2CP, a derivative of 4-*O*-benzoyl-3-methoxy-beta-nitrostyrene, binds to the CLEC-2 and inhibits the platelet aggregation and cancer metastasis in vivo [118]. Phosphodiesterases (PDEs) regulate cyclic nucleotide signaling by catalyzing cyclic adenosine monophosphate (cAMP) and cyclic guanosine monophosphate (cGMP) to the inactive form. PDE2, PDE3, and PDE5 expression is detected in platelets [119,120]. Selective PDE inhibitors, such as caffeine and theophylline, inhibit platelet aggregation and cancer cell invasion, and enhance anti-cancer drug efficiency in vivo [121,122,123]. Glycoprotein IIb/IIIa (GPIIb/IIIa) antagonists inhibit platelet aggregation, and a pre-clinical study shows that treatment with GPIIb/IIIa antagonists significantly decreases tumor nodules in lung metastasis [124].

### 4.2. Targeting Platelets Is a Potential Strategy to Overcome Drug Resistance

The chemotherapeutic response in human epidermal growth factor receptor 2-negative breast cancer is significantly associated with that platelets surround primary tumor [125]. Clinical data show that primary tumor cells surrounded with platelets are less responsive to neo-adjuvant chemotherapy. In addition, platelets promote EMT in cancer cells, which is associated with chemoresistance [126,127]. It has been reported that platelet-derived ADP and ATP increase the level of EMT inducer Slug and cytidine deaminase, and enhances gemcitabine resistance. The P2Y12 inhibitor abolishes the survival signal induced by platelet-derived ADP and ATP [39]. These data suggest that targeting platelets is a potential strategy to overcome chemoresistance.

### 4.3. Platelets in Anti-Cancer Drug Delivery

Platelets, and their secreted vesicles, are potential drug delivery vehicles [128]. Platelets have little effect on drug activity, and using them as drug delivery vehicles may reduce side effects [129]. The platelet-loaded drugs are protected from clearance and, thus, can circulate in blood for a relatively long time [130]. A recent study shows that platelet membrane-coated particles specifically deliver drugs to CTC and reduce cancer lung metastases in mice [131]. Current drug delivery systems depend on unique cancer markers and tumor-specific microenvironment cues, such as pH and hypoxia. However, the microenvironment of CTCs is different from the solid tumor. Platelets may provide an effective delivery system to target CTCs and inhibit cancer metastasis.

## 5. Conclusions and Future Direction

**Figure 1:**
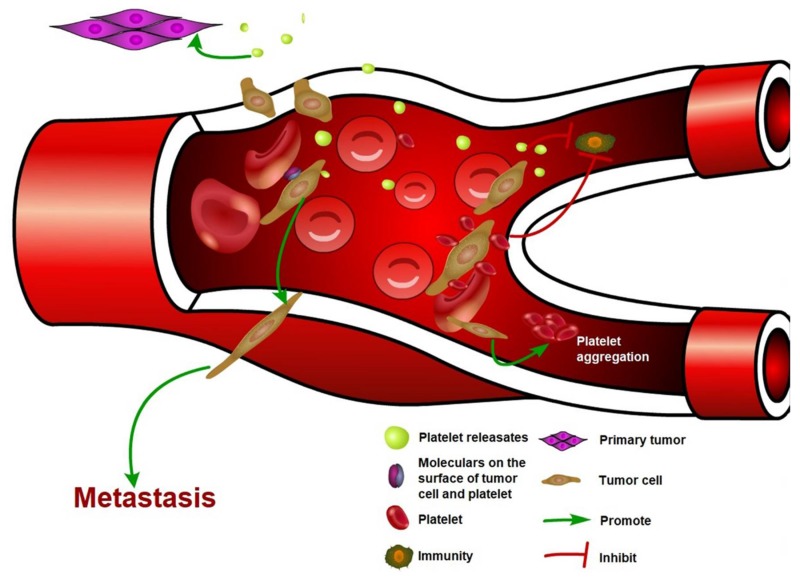
The interaction between cancer cell and platelet. Circulating tumor cells induce platelet activation and aggregation. Activated platelets release a variety of factors, which promote primary tumor growth and cancer metastasis. Binding of platelets also protects CTCs from flow shear force and immune cell attacks.

Platelet-cancer cell interaction promotes cancer cells metastasis by enhancing CTC survival and extravasation (Figure 1). Growth factors, metabolites, and microRNA released by activated platelets induce EMT and enhance cancer cell stemness, which is crucial for cancer cell colonization at the distant organs (Table 1). Importantly, cancer cells also induce platelet activation and aggregation, and subsequently elevate the risk of thrombosis. Therefore, targeting platelet-cancer cell interaction is a potential strategy to reduce both cancer metastasis and cancer-associated thrombosis. Nevertheless, targeting platelets has not been utilized for cancer therapy in the clinic because the cancer cell-platelet interaction is still not completely understood. For instance, the key factor that regulates cancer cell-platelet interaction has not been identified; roles of platelets in tumor initiation and primary tumor development remained to be determined. We believe that addressing these questions may help to achieve the goal of targeting platelet-cancer interaction for cancer therapy.

## Figures and Tables

**Table 1:** Function of platelet–derived factors and proteins in cancer development and progression.

Platelet Related Factors	Function	Mechanism	Inhibitors	Ref
TGF-β	Promote primary tumor growth,	TGF-β1 promotes cancer cell proliferation directly	SB431542, decorin	[46,47]
	Enhance EMT phenotype and promote tumor cell extravasation	TGF-β releasing induces the EMT phenotype depending on podoplanin		[70,71]
		Platelets and tumor cells contacts activate TGF-β/SMAD and NF-κb pathway		
	Downregulate reactivity of NK cell, inhibit antitumor immunity	TGF-β down-regulates the NKG2D expression, the activating immunoreceptor		[95,96]
		TGF-β downregulates inflammatory cytokine production		
VEGF	Promote the angiogenesis	Enhance endothelial cell growth		[52]
PDGF	Promote the tumorigenesis	Stimulate the cells in tumor stroma and promote angiogenesis	Olaratumab, imatinib, sunitinib, sorafenib, pazopa-nib, nilotinib, cediranib, trapidil	[53]
	Induce EMT markers	Upregulate the expression of COX-2		[76]
PF4	Inhibit tumor growth and metastasis	Inhibit endothelial proliferation in vitro and angiogenesis in vivo		[57]
	Promote Kras-driven tumorigenensis	Promote platelet production and modulate the tumor mocroenvironment to accelerate the tumor growth		[55]
P2Y12	Promote primary tumor growth	Recruits Gβγ subunits, causing phosphoinositide-3-kinase- dependent Akt phosphorylation and Rap1b activation	clopidogrel, ticagrelor, prasugrel	[40,41]
		Induce ERK1/2 and paxillin Ser83 phosphorylation		
MiRNA 24	Induce the tumor growth inhibition at early stage	Transfer to tumor cells, then induce the mitochondrial dysfunction and tumor cell apoptosis		[60]
MiRNA 939	promotes epithelial to mesenchymal transition	Transfer to tumor cells, downregulate E-cadherin and up-regulate vimentin		[72]
CLEC2	Promote EMT and tumor extravasation in mouse model	Bind with Aggrus, attenuate Aggrus-induced platelet aggregation	2A2B10, 2CP	[73]
Integrin (α6β1, αIIbβ3)	Promote metastasis	Bind with molecular on tumor cell surface, such as ADAM9	ML464, scFv Ab; A11, 7E3 F(ab’)2	[79,80]
LPA	Enhance bone metastasis	enhances the LPA-dependent production of IL-6 and IL-8 to stimulate osteoclast-mediated bone resorption		[81]
Asm	Promote tumor cell adhesion and metastasis	Activate α5β1 on melanoma cells		[88]
Ask1	Promote cancer metastasis	Protect the cancer cells from anoikis		[86,87]

## Abbreviations

VEGF	Vascular endothelial growth factor
PDGF	Platelet derived growth factor
P2Y12	Platelet factor 4 (PF4), ADP receptor
CLEC2	C-type lectin-like receptor 2
LPA	Lysophosphatidic acid
Asm	Acid sphingomyelinase
Ask1	Apoptosis signal-regulating kinase 1
